# Implementation of a pre-calving vaccination programme against rotavirus, coronavirus and enterotoxigenic *Escherichia coli* (F5) and association with dairy calf survival

**DOI:** 10.1186/s12917-022-03154-2

**Published:** 2022-01-28

**Authors:** Dagni-Alice Viidu, Kerli Mõtus

**Affiliations:** Institute of Veterinary Medicine and Animal Sciences, Estonian University of Life Sciences, Kreutzwaldi 62, 51006 Tartu, Estonia

**Keywords:** Lactogenic immunity, Vaccination practices, Dairy calf, Mortality, Estonia

## Abstract

**Background:**

Diarrhea is one of the most common diseases and causes of death in calves during the first month of life. Pre-calving vaccination programme (PVP) against the most common diarrhea-causing pathogens could help to avoid this threat if hyperimmune transition milk (TM) is fed to calves throughout the whole susceptibility period. The aim of this retrospective cohort study was to reveal the implementation practices of PVPs in large commercial dairy farms and to compare calf-level mortality hazards during the first year of vaccination (V+ period) and a year before implementing the vaccination programme (V- period). A questionnaire was filled out in 15 large-scale dairy farms in Estonia that used PVP. The farms were assigned into three groups based on compliance with the vaccine directions for use and TM feeding practices. Calf-level time-to-event data was analyzed with an observation period of 21 days and on-farm mortality due to diarrhea being the event of interest.

**Results:**

During the V+ period, a significant decline in diarrhea-induced calf mortality was identified in three out of six herds that followed vaccination instructions and fed TM for at least 14 days. On average, calf mortality hazard due to diarrhea decreased among these herds (hazard rate ratio (HR) = 0.72, 95% confidence interval (CI) 0.63; 0.81). In the group of correctly vaccinating herds where TM was fed for less than 14 days, diarrhea-induced calf mortality decreased in two herds and remained unchanged in two herds with average diarrhea-induced calf mortality hazard declining significantly during the vaccination period (HR = 0.24, 95% CI 0.14; 0.41). Among the three farms that deviated from the vaccination instructions, the average calf mortality hazard increased in the V+ period (HR = 1.61, 95% CI 1.21; 2.14).

**Conclusions:**

This study revealed that implementing a PVP might aid to reduce diarrhea-induced calf mortality in large commercial dairy farms. There is a need to increase veterinarians´ and farmers´ awareness about the importance of including pregnant heifers into the vaccination programme and emphasize the importance of prolonged feeding of hyperimmune TM to calves.

**Supplementary Information:**

The online version contains supplementary material available at 10.1186/s12917-022-03154-2.

## Background

Diarrhea and other digestive problems are one of the most prevalent diseases in pre-weaned dairy calves and cause more than half of deaths during the first months of life [1–4]. Neonatal calf diarrhea occurs mostly during the first 21 days of life with peak morbidity before two weeks of age [5–8]. *Escherichia coli*, bovine rotavirus, bovine coronavirus and *Cryptosporidium* spp. are among the most common pathogens known to cause diarrhea in neonatal calves [9, 10]. Enterotoxigenic *Escherichia coli* (ETEC) affects calves primarily during the first week of life, whereas rotavirus, coronavirus and *Cryptosporidium* spp. are common causes of diarrhea during the first two to three weeks of calves’ life [7, 9–12].

During evolution, bovines forfeited the ability to transmit immunoglobulins prenatally while immediate postnatal transfer is well developed [13]. As calves are born agammaglobulinemic, their survival and resilience to infections during the first weeks of life depend largely on the immunoglobulins acquired from their feed [14–16]. Maternally derived antibodies are absorbed by the calf by means of non-selective protein transfer through the epithelium of the small intestine [13, 17]. Protein absorption occurs most effectively during the first 12 hours after birth and ceases almost entirely by 24 hours, after which the absorbed antibodies are continuously reabsorbed back into the intestinal tract in small quantities [15, 18]. The majority of antibodies that are ingested after cessation of protein transmission will have to function on the surface of enterocytes or in the gut lumen [18], with the latter being the principle of lactogenic immunity. Endogenous production of antibodies starts shortly after birth, but during the first weeks of life, the amount of immunoglobulin G produced by the calf is insufficient to reach a plasma concentration of at least 10 g/l [15], a value that is commonly considered the cut-off point for adequate immunological protection.

The essence of lactogenic (also referred to as colostral) immunity is to ensure that pathogen-specific antibodies are constantly present in the gut lumen in high quantities and can neutralize the pathogen rapidly. Previous studies have identified an increase in antibody titers in dam serum, colostrum and milk as well as calf serum due to pre-calving vaccination of the dam against ETEC, rotavirus and coronavirus, among others [19–22]. Vaccinating dry cows during the last trimester of gestation increases the level of these pathogen-specific antibodies in the colostrum and milk for up to 28 days or more and enhances the nutritional value of colostrum [23–25]. To maximize the potential benefit gained from pre-calving vaccination, hyperimmune milk or colostrum must be fed to calves for the entire susceptible period of the targeted pathogen [8, 19, 20, 26].

Although the importance of colostrum on calf health and survival has been studied for over a century [27, 28], it has mostly focused on colostral transfer and uptake of immunoglobulins, whereas less attention is paid on the ways to maximize the potential benefit gained from it. Implementing pre-calving vaccination (PV) and taking advantage of the enhanced lactogenic immunity could be a useful tool for reducing morbidity and mortality due to diarrhea. Previous studies have demonstrated that PV is efficient in decreasing the incidence, severity and duration of diarrhea and pathogen shedding in calves [12, 29–32]. Regarding the effect on calf mortality, early studies on this subject showed varying results [28, 29, 33, 34], and studies conducted within this century have not reached a consensus either [21, 31, 35].

The majority of earlier studies focusing on PV have used different antigens or their combinations or simultaneously applied other preventive remedies, but the effect of merely vaccinating against rotavirus, coronavirus and ETEC is inconclusive [21, 31, 33–37]. Only one field trial [29] has been published that analyzed the effect of PV against rotavirus, coronavirus and ETEC on calf mortality and used a similar methodology regarding control group selection, but that study included too few cases to make reliable conclusions about the effect of PV on calf mortality. Other studies regarding PV and its impact on calf mortality have either been small-scale or designed as experimental studies. Additionally, the results of many former studies are regrettably not applicable in modern dairy farms because of major differences in the housing- and management systems between older or small-scale farms and large commercial farms. Another concern is the shortage of knowledge about the importance of calf feeding practices on the outcome of PV as no studies have been done to describe or to analyze the effect of colostrum and transition milk (cow´s milk in the first four post-partum days, TM) feeding regimes on the outcome of a vaccination programme. Because of the scarcity of information, the current study was conducted with an aim to investigate the implementation of a pre-calving vaccination programme (PVP) against bovine rotavirus, bovine coronavirus and enterotoxigenic *E. coli* (F5 antigen) on herd level and to analyze the possible association between different calf feeding regimes and calf survival in large-scale commercial dairy farms.

## Results

A total of 15 dairy farms were enrolled in the present study. Study farms were categorized based on vaccination and calf feeding practices and formed three groups - complete extended pre-calving vaccination programme users (CEU), complete standard pre-calving vaccination programme users (CSU) and incomplete pre-calving vaccination programme users (ICU). Data regarding the year before implementing the vaccination programme (V- period) and the first year of vaccination (V+ period) was collected and analyzed about each herd.

### Farm data

**Fig. 1:**
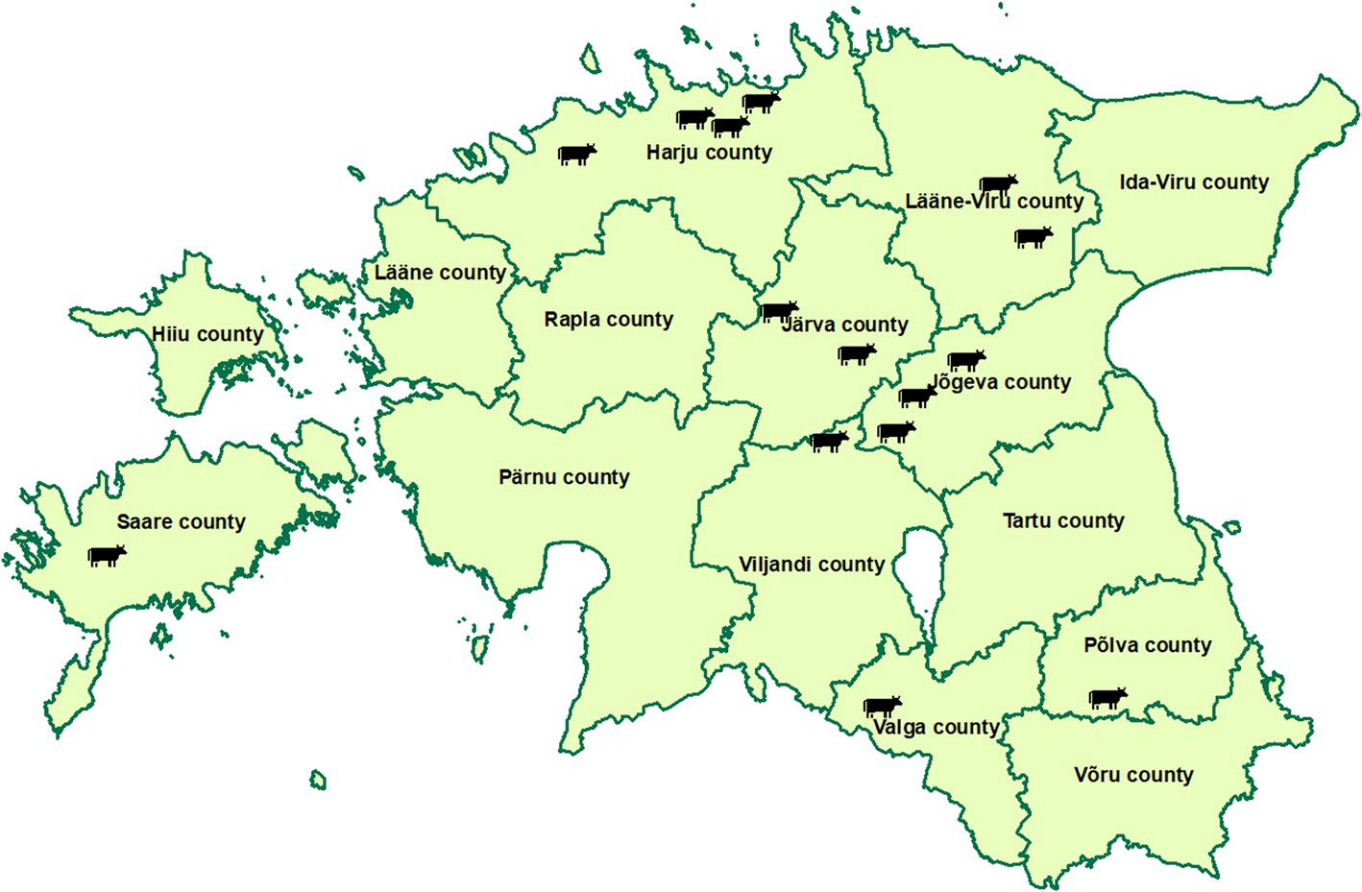
Location of the 15 study farms in Estonia

Average herd size was 711 cows and the average 305-day milk yield per cow in 2019 was 10,344 liters. Individual data about each study farm is presented in Table 1 and the location of the 15 study herds is shown in Fig. 1.

**Table 1:** Descriptive information about 15 Estonian dairy herds implementing a pre-calving vaccination programme

Farm ID	Study group^a^	Herd size^b^	Average milk yield per cow (kg)	Start of vaccination	Calves born during V- period^c^ (n)	Calves born during V+ period^d^ (n)
1	CSU	449	10399	31.01.2018	NA^e^	NA^e^
2	ICU	238	10768	31.10.2017	199	235
3	CEU	2443	12548	28.07.2016	2560	2619
4	CEU	513	9803	30.11.2017	543	540
5	CEU	1696	10246	05.09.2018	1877	2426
6	CSU	734	11116	25.07.2018	NA^e^	NA^e^
7	CEU	964	10496	21.12.2016	791	1057
8	CSU	607	10557	21.11.2016	670	645
9	ICU	648	10537	31.05.2015	784	749
10	CSU	301	10424	15.10.2018	471	577
11	ICU	423	9057	25.10.2017	378	334
12	CEU	437	8821	26.07.2018	484	417
13	CEU	713	11953	14.02.2017	505	791
14	CSU	268	9984	14.03.2017	259	313
15	CSU	238	8445	02.07.2017	284	287

^a^*CEU* complete extended pre-calving vaccination programme user, *CSU* complete standard pre-calving vaccination programme user, *ICU* incomplete pre-calving vaccination programme user

^b^Number of cows at 31.12.2019

^c^V- period – a year before implementing the vaccination programme

^d^V+ period – the first year of vaccination

^e^NA- data not available, farm was not included in the mortality analysis

All included herds were loose-housed farms. Four farms (27%) reared their calves in individual pens for at least the first 21 days, 20% of farms (*n* = 3) provided this housing type for 14 days and the rest of the farms (*n* = 8) had diverse lengths of individual keeping of calves, ranging from three to nine days, after which the calves were moved to group pens of varying size. The persons answering the questions during the farm visit or phone interview were mainly veterinarians (*n* = 11), although some of them also fulfilled the obligations of a farm manager. Three respondents (20%) were farm managers and one was a farm owner.

### Implementation of pre-calving vaccination

The most common incentives for vaccination were to decrease the mortality and lower the incidence of diarrhea among calves (80% and 73% of the farms, respectively). All farms had carried out diagnostic tests prior to vaccination to determine the causative agents of calf diarrhea, and rotavirus (73% of farms) and *Cryptosporidium* spp. (60% of farms) were the most commonly identified pathogens (Table 2).

**Table 2:** Motivation for initiating vaccination programme and identified diarrhea-causing pathogens in 15 Estonian dairy herds implementing a pre-calving vaccination programme

Variable	Category	Farm number															Total (n)	% of farms
		1	2	3	4	5	6	7	8	9	10	11	12	13	14	15		
Expectations for pre-calving vaccination	Decrease the incidence of calf diarrhea			x	x	x		x	x	x	x	x	x	x	x		11	73%
	Decrease calf mortality	x	x	x		x	x	x		x		x	x	x	x	x	12	80%
	Reduce the severity of calf diarrhea	x				x			x		x						4	27%
	Lower treatment costs for calf diarrhea								x				x				2	13%
	Improve calf weight gain													x			1	7%
Diarrhea-causing pathogens diagnosed before the start of pre-calving vaccination	Rotavirus	x		x	x	x	x	x	x	x	x	x			x		11	73%
	Coronavirus			x						x	x				x		4	27%
	*Cryptosporidium* spp.			x	x	x		x	x	x	x	x			x		9	60%
	ETEC^a^				x					x			x			x	4	27%
	*Salmonella* Dublin							x									1	7%
	No information^b^		x											x			2	13%

^a^*ETEC* Enterotoxigenic *Escherichia coli*

^b^The respondent could not state the exact pathogens that were identified

In all study herds, pre-calving vaccination was conducted 3-12 weeks before the expected calving date as was recommended by the vaccine manufacturers. Four farms did not vaccinate their pregnant heifers, but in one of those farms, milk from primiparous cows was not fed to the calves. Detailed information about the implemented vaccination protocols is presented in Table 3.

**Table 3:** Details of the pre-calving vaccination protocols implemented in 15 Estonian dairy herds

Variable	Category	Farm number															Total (n)	% of farms
		1	2	3	4	5	6	7	8	9	10	11	12	13	14	15		
Vaccinated animal groups	Cows and pregnant heifers	x		x	x		x	x	x		x		x	x	x	x	11	73%
	Only cows		x			x^a^				x		x					4	27%
Executor of the vaccinations	Veterinarian					x		x	x		x	x	x	x	x		8	53%
	Veterinary technician^b^		x													x	2	13%
	Veterinarian and veterinary technician^b^			x	x		x			x							4	27%
	Farm owner	x															1	7%
Products used during the first year of vaccination	Bovigen Scour			x		x	x				x		x				5	33%
	Rotavec Corona							x	x	x				x	x		5	33%
	Both	x	x		x							x				x	5	33%
Region of administration	Neck region			x		x	x		x	x	x	x	x		x		9	60%
	Gluteal region	x	x		x			x			x				x	x	7	47%
	Shoulder region													x			1	7%

^a^did not feed milk from primiparous cows

^b^veterinary technician or veterinary assistant

### Colostrum and transition milk feeding practices

In most of the farms (*n* = 14, 93%), the first colostrum meal was fed to the calves within the first two hours after birth, and the minimal amount of colostrum fed ranged from 2 – 4 liters. The duration of feeding calves TM from vaccinated cows differed from 1-90 days. Six farms fed only TM of vaccinated cows during the first 14 days of calves’ life while other farms combined different types of feeds during this period (Table 4).

**Table 4:** Calf feeding practices in 15 Estonian dairy herds implementing a pre-calving vaccination programme

Variable	Category	Farm number															Total (n)	% of farms
		1	2	3	4	5	6	7	8	9	10	11	12	13	14	15		
Time of the first colostrum feeding (hours after birth)	<1	x		x	x	x									x	x	6	40%
	1-2						x	x	x	x	x	x	x	x			8	53%
	>4		x														1	7%
Amount of colostrum fed during the first feeding (liters)	2		x		x				x				x	x			5	33%
	2.5						x										1	7%
	3	x		x						x	x	x			x	x	7	47%
	4					x		x									2	13%
Method of feeding the first colostrum	Nipple bottle	x	x		x		x		x	x	x	x	x	x	x	x	12	80%
	Esophageal tube			x	x	x		x			x	x		x			7	47%
	Suckling		x														1	7%
Calf milk feeds during the first 14 days	TM^a^			x	x	x		x					x	x			6	40%
	TM^a^ and MR^b^								x	x	x	x				x	5	33%
	TM^a^ and mix of TM^a^/MR^b^	x															1	7%
	TM^a^ and BTM^c^														x		1	7%
	TM^a^ and WM^d^ or mix of WM^d^/MR^b^		x				x										2	13%
Duration of feeding TM^a^	Days	4	7	14	21	14	7	90	6	2	7	1	14	30	4	3	-	-

^a^*TM* transition milk (milk from cows up to four days in milk)

^b^*MR* milk replacer

^c^*BTM* bulk tank milk (saleable milk)

^d^*WM* waste milk (mastitic or high somatic cell count milk or milk containing traces of antibiotics, all unsuitable for retail)

### Farmers’ opinions about the effect of pre-calving vaccination programme

Majority of the farmers from CEU and CSU herds suggested that during the vaccination period, the incidence of calf diarrhea and calf mortality reduced (67% and 83% of CEU herds, and 83% and 67% of CSU herds, respectively). None of the respondents suggested an increase in calf diarrhea incidence or mortality after the start of the PVP. In total, 83% of the respondents from CEU herds (*n* = 5), 50% from CSU herds (*n* = 3) and one of the three ICU herds thought that implementing a PVP is economically profitable, while one CEU, two CSU and four ICU herd representatives were uncertain and one interviewee from the CSU group suggested that the vaccination is not cost-effective (Table 5).

**Table 5:** Interviewees’ opinion about the effect and cost-effectiveness of pre-calving vaccination programme in 15 Estonian dairy farms

Variable	Category	Farm number															Total (n)	% of farms
		1	2	3	4	5	6	7	8	9	10	11	12	13	14	15		
Incidence of diarrhea	Decreased	x		x		x			x	x	x		x	x	x	x	10	67%
	Did not change						x										1	7%
	Hard to say		x		x			x				x					4	27%
Calf mortality	Decreased	x		x	x	x				x	x		x	x	x	x	10	67%
	Did not change						x		x			x					3	20%
	Hard to say		x					x									2	13%
Economic profitability of the vaccination	Cost-effective			x	x	x			x	x			x	x	x	x	9	60%
	Not cost-effective						x										1	7%
	Hard to say	x	x					x			x	x					5	33%

### Association between implementing a pre-calving vaccination programme and diarrhea-induced calf mortality

Based on the vaccination procedure and TM feeding practices, six herds met the requirements of CEU, four farms were classified as CSU and three as ICU. The number of calf-level observations included in the analysis was 14,610 in CEU group, 3,506 in CSU group and 2,679 in ICU group. Herd-level Kaplan-Meier survival graphs for all CEU, CSU and ICU farms can be found in Supplementary Figs. 1, 2 and 3, respectively.

Yearly herd-level diarrhea-induced on-farm calf mortality rates ranged between 5.06 and 17.77 per 100 calf-months in CEU farms in the V- period. In three out of six CEU farms calf mortality hazard (CMH) decreased significantly during the V+ period compared to V- period. In two CEU farms CMH was higher in the V+ period, and in one CEU farm, no significant change in CMH was identified (Table 6, Supplementary Fig. 1). On average across six CEU farms, CMH decreased during the first year of vaccination compared to pre-vaccination period (hazard rate ratio (HR) = 0.72, 95% CI 0.63; 0.81, *p* < 0.001) according to the mixed-effects Cox regression model (herd included as random effect). The herd-level average age at death ranged from 9.4 to 13.8 days and from 9.2 to 13 days across the six CEU herds during the V- and V+ periods, respectively. According to the linear mixed-effect regression model, there was no statistically significant difference between the calf-level age at death in V- and V+ period in CEU herds (Coef = 0.006, 95% CI -0.001; 0.002, *p* = 0.401).

**Table 6:** Mortality rates and the average age at death among calves up to 21 days of age, during the first year of vaccination (V+) and a year before implementing the vaccination programme (V-) and the associated mortality hazards in 13 Estonian dairy herds implementing a pre-calving vaccination programme

Farm ID^a^	Overall mortality rate^c^ (V-)	Diarrhea-induced mortality rate^d^(V-)	Proportion of deaths due to diarrhea (V-)	Mean age at death^e^ (V-)	Overall mortality rate^c^ (V+)	Diarrhea-induced mortality rate^d^ (V+)	Proportion of deaths due to diarrhea (V+)	Mean age at death^e^ (V+)	Overall HR^f^	*p*-value^f^	Diarrhea-induced HR^g^	*p*-value^g^
CEU farms^b^												
3	15.22 (13.38; 17.31)	12.92 (11.24; 14.86)	84.9%	11.57	5.42 (4.38; 6.69)	1.07 (0.67; 1.72)	19.7%	11.91	0.36 (0.28; 0.46)	<0.001	0.08 (0.05; 0.14)	<0.001
4	11.91 (8.73; 16.23)	5.06 (3.15; 8.14)	42.5%	11.09	6.08 (3.92; 9.43)	3.95 (2.30; 6.81)	65.0%	12.96	0.51 (0.30; 0.87)	0.013	0.77 (0.37; 1.58)	0.476
5	13.95 (11.91; 16.35)	12.59 (10.65; 14.87)	90.3%	13.83	5.95 (4.83; 7.32)	4.68 (3.70; 5.91)	78.7%	12.67	0.42 (0.32; 0.55)	<0.001	0.37 (0.28; 0.49)	<0.001
7	41.17 (35.62; 47.59)	17.77 (14.26; 22.16)	43.2%	10.08	46.18 (41.06; 51.94)	45.35 (40.28; 51.06)	98.2%	11.25	1.10 (0.92; 1.33)	0.298	2.51 (1.95; 3.23)	<0.001
12	11.61 (8.29; 16.25)	10.24 (7.16; 14.65)	88.2%	9.37	21.68 (16.61; 28.31)	19.27 (14.53; 25.58)	88.9%	9.19	1.85 (1.20; 2.83)	0.005	1.86 (1.18; 2.93)	0.008
13	13.48 (9.94; 18.33)	13.16 (9.66; 17.95)	97.6%	10.00	4.79 (3.21; 7.14)	4.39 (2.89; 6.67)	91.6%	10.63	0.36 (0.22; 0.60)	<0.001	0.34 (0.20; 0.57)	<0.001
CSU farms^b^												
8	0.71 (0.23; 2.19)	0.47 (0.12; 1.88)	66.2%	2	0.74 (0.24; 2.29)	0.49 (0.12; 1.97)	66.2%	12.5	1.04 (0.21; 5.15)	0.963	1.04 (0.15; 7.39)	0.968
10	8.19 (5.44; 12.33)	5.34 (3.22; 8.86)	65.2%	10.17	3.45 (1.96; 6.08)	2.88 (1.55; 5.34)	83.5%	12.60	0.42 (0.21; 0.84)	0.014	0.53 (0.24; 1.19)	0.123
14	20.05 (14.10; 28.51)	17.46 (11.97; 25.46)	87.1%	7.69	4.15 (2.07; 8.29)	1.55 (0.50; 4.82)	37.3%	10.17	0.21 (0.09; 0.45)	<0.001	0.09 (0.03; 0.29)	<0.001
15	18.32 (12.81; 26.20)	11.60 (7.40; 18.19)	63.3%	9.66	6.31 (3.50; 11.40)	1.72 (0.56; 5.34)	27.3%	8.50	0.34 (0.17; 0.68)	0.002	0.15 (0.04; 0.50)	0.002
ICU farms^b^												
2	0.80 (0.11; 5.67)	0.80 (0.11; 5.67)	100.0%	19.5	1.33 (0.33; 5.33)	0.67 (0.09; 4.73)	50.4%	17.5	1.68 (0.15; 18.52)	0.672	0.85 (0.05; 13.54)	0.906
9	20.77 (16.73; 25.79)	16.72 (13.13; 21.28)	80.5%	8.45	15.70 (12.38; 19.91)	13.39 (10.35; 17.32)	85.3%	11.09	0.75 (0.54; 1.03)	0.075	0.79 (0.56; 1.13)	0.199
11	5.90 (3.49; 9.96)	4.21 (2.27; 7.83)	71.4%	7.3	40.95 (32.61; 51.43)	33.76 (26.26; 43.83)	82.4%	9.28	6.77 (3.82; 12.00)	<0.001	7.77 (3.98; 15.17)	<0.001

^a^Farms 1 and 6 were excluded from the mortality analyses due to late ear-tagging of calves

^b^CEU – complete extended pre-calving vaccination programme user, CSU – complete standard pre-calving vaccination programme user, ICU – incomplete pre-calving vaccination programme user

^c^Overall number of deaths per 100 calf-months with 95% confidence intervals

^d^Number of deaths due to diarrhea per 100 calf-months with 95% confidence intervals

^e^Mean age at death due to diarrhea in days

^f^Hazard rate ratio for overall calf mortality in V+ period compared to V- period in Cox random-effect regression model with 95% confidence interval and the associated *p*-value

^g^Hazard rate ratio for diarrhea-induced calf mortality in V+ period compared to V- period in Cox random-effect regression model with 95% confidence interval and the associated *p*-value

In CSU farms, the yearly diarrhea-induced calf mortality rate ranged between 0.47 and 17.46 per 100 calf-months in the V- period. In two out of four CSU farms CMH decreased significantly during the V+ period compared to V- period. In the other two CSU farms no significant change in CMH was identified during the V+ period (Table 6, Supplementary Fig. 2). Across the four CSU farms, CMH decreased during the V+ period compared to V- period (HR = 0.24, 95% CI 0.14; 0.41, *p* < 0.001). The average age at death ranged from 2.0 to 10.2 days during the V- period and between 8.5 and 12.6 days during the V+ period across the four CSU herds. According to the linear mixed-effect regression model, the increase in calf age at death in V+ period compared to V- period was significant (Coef = 0.006, 95% CI 0.001; 0.012, *p* = 0.018).

**Fig. 2:**
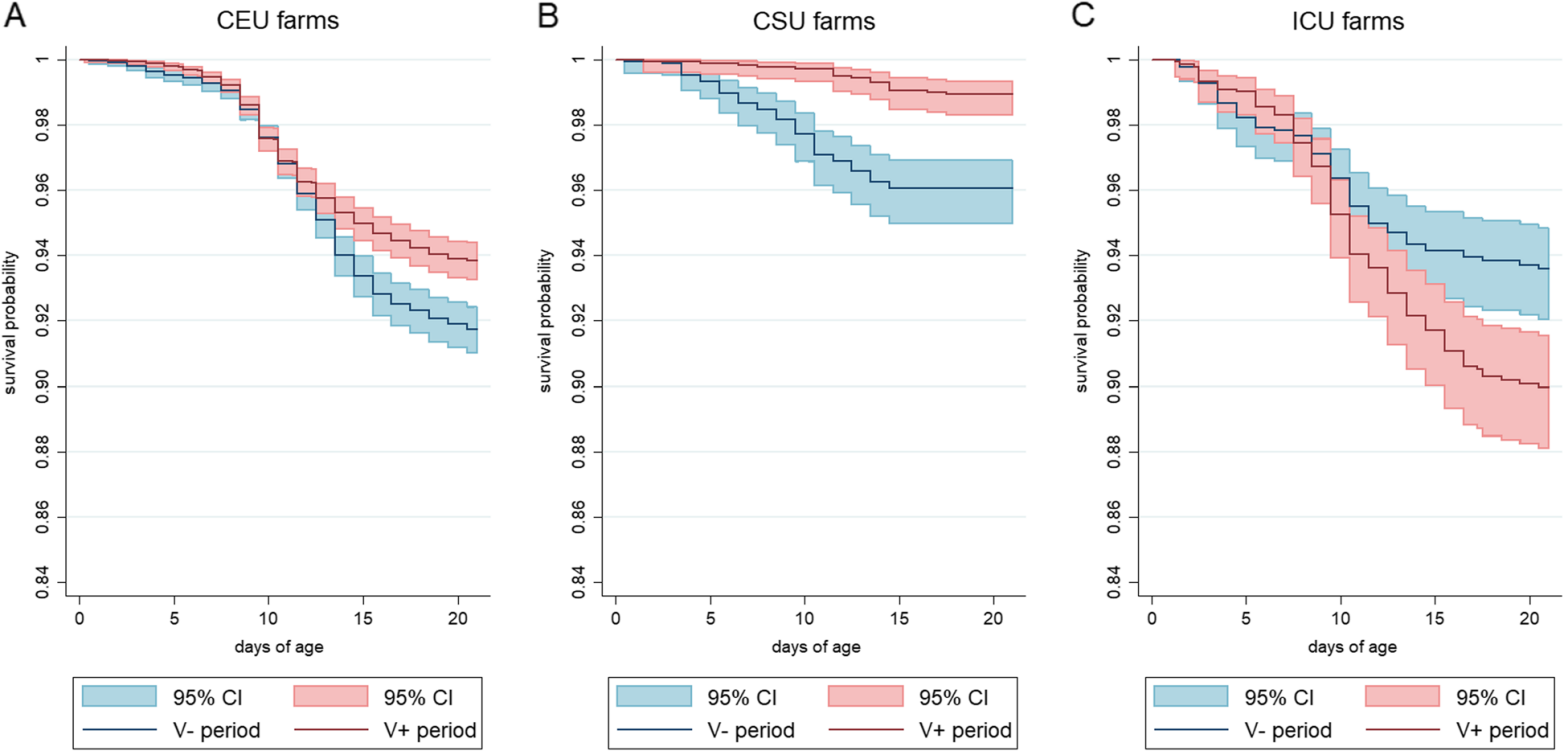
Kaplan-Meier survival graphs presenting diarrhea-induced mortality probabilities of calves up to 21 days of age in (**A**) six farms using the complete extended vaccination programme (CEU farms), **B** four farms using the complete standard vaccination programme (CSU farms) and **C** three farms using the incomplete vaccination programme (ICU farms) during the first year of vaccination (V+ period) and a year before implementing the vaccination programme (V-period)

In ICU farms, the yearly diarrhea-induced calf mortality rate ranged between 0.80 and 16.72 per 100 calf-months in the V- period. In two out of three ICU farms, compared to the V- period, no significant change in the calf mortality rate was identified during the V+ period and in one ICU farm there was a significant increase in CMH during the V+ period (Table 6, Supplementary Fig. 3). Across the three ICU farms, CMH increased significantly during the V+ period (HR = 1.61, 95% CI 1.21; 2.15, *p* = 0.001). The average age at death ranged from 7.3 to 19.5 days during the V- period and between 9.3 and 17.5 days during the V+ period across the three ICU herds. Compared to the V- period, the average calf age at death increased significantly at V+ period (Coef = 0.007, 95% CI 0.003; 0.01, *p* < 0.001). Calf survival distribution in V- and V+ period across all CEU, CSU and ICU farms is illustrated in Fig. 2. According to the Kaplan-Meier curve, the difference in diarrhea-induced calf mortality in the two study periods became apparent after 10-12 days of calf age in CEU herds, whereas in CSU herds the difference was visible from 4-5 days of age.

## Discussion

To the authors’ knowledge, this is the first field study providing an overview of on-farm implementation practices of a pre-calving vaccination programme against bovine rotavirus, bovine coronavirus and ETEC which also analyzed the possible association between implementation practices of PVP and calf mortality in large commercial dairy farms.

### Implementation of pre-calving vaccination

The decision to start the vaccination programme was mostly driven by the desire to decrease calf mortality and incidence of diarrhea and less commonly to increase weight gain of the calves and to decrease the severity of the diarrhea. Herd-level calf mortality rates of the vaccinating herds in the V- period differed greatly, referring to individual and highly variable thresholds for taking specific disease prevention measures into use. Prior to vaccination, most of the study farms had confirmed the presence of one or more pathogens included in the vaccine, meaning that the decision to start the vaccination programme was mostly well elaborated and justified.

During the first year of implementing the vaccination programme, an equal number of farms used either only Rotavec Corona (RC), only Bovigen Scour (BS) or both, but all farms starting with RC changed to BS later due to limited availability of RC (data not shown). In all farms, cattle were vaccinated between 3-12 weeks before expected calving as was suggested by the vaccine manufacturers; however, in three farms, pregnant heifers were not vaccinated while feeding TM of primiparous cows to calves still occurred. Arranging vaccination at the time window of 3-10 weeks before calving has proven to be more effective in raising antibody titers in calf’s serum than vaccination less than three or more than 10 weeks before parturition [36, 38]. In all farms except for one, the persons carrying out the vaccinations had some level of veterinary education. Conducting any kind of veterinary procedure without appropriate training might induce discrepancies in the execution of the procedure, but the farm under question in this study was excluded from the mortality analysis because of late ear-tagging of the calves, thus not affecting the study results.

The vaccine administration sites differed remarkably across the study farms. Although the SPC of BS does not suggest a specific area for vaccine administration, the SPC of RC recommends administering the vaccine into the neck region [39, 40] which was used as the only possible injection site in only seven herds. The region of administration might sometimes influence the effectiveness of the drug [41, 42]; however, according to the authors´ knowledge, it has not been investigated whether this is important regarding the specific vaccines used in this study. Neck region was used for injection of the vaccine in all study groups and among users of either of the two products; however, shoulder and gluteal regions were also used, indicating that veterinarians either do not follow the vaccination instructions accurately or choose the administration route that is most convenient to use in loose-housed systems.

### Calf feeding practices

Colostrum provides calves with systemic immunity that is vital for survival and resistance to different infections during the first days of life. Without proper colostrum feeding practices, the following measures have limited capability in preventing diseases and death. As was identified in this study, most of the farms followed the recommended colostrum feeding practices [43, 44]; however, as we did not measure the IgG concentration in either colostrum or calf serum, this cannot confirm adequate systemic immunity of the calves.

The mortality and outcome of a PVP is probably considerably affected by the length of the subsequent TM feeding, which protects calves essentially against rota- and coronavirus replication in the gut lumen via the presence of lactogenic antibodies that are mainly of IgG1 type [13, 45]. Many authors have pointed out the need to continue feeding colostrum or TM for longer periods than only the first day of calf’s life [19, 20, 26, 29], yet this is rarely seen in earlier studies regarding PVPs. In addition to protection from diseases and death, prolonged colostrum and TM feeding can reinforce cellular immune responses for vaccinations later in life and enhance the weight gain and development of intestinal villi of young calves [46–49]. Studies conducted with beef cows have shown that pre-calving vaccination induces strong lactogenic immunity and protects calves effectively against diarrhea [33, 37, 50]. This effect can be attributed to the fact that calves are usually allowed to suckle their dams at least during the whole risk period of two weeks and therefore have a good level of protective lactogenic immunity [23, 30], in contrast to large-scale dairy farms, where it is rather unusual to feed calves whole milk for such a long period.

Instructions regarding the most beneficial calf feeding practices varied greatly between the two vaccines that were used in the study farms. The summary of product characteristics (SPC) of RC states that the protection of the calves against diarrhea-causing pathogens depends on the presence of colostral antibodies in their gut lumen during the first two to three weeks of life and highlights the need to feed hyperimmune milk throughout the whole mentioned period [40]. The SPC of BS only emphasizes the need to feed calves with a sufficient amount of colostrum during the first days of life [39]. Despite the presumption that herds that started vaccination with BS might fall into the category of CSU farms because of applying shorter duration of TM feeding as is suggested by the SPC of BS, this was not the case in this study. Durel and colleagues [22] compared the potential of RC and BS to increase specific antibody levels in cow and calf serum and colostrum and demonstrated that neither vaccine is inferior to the other, also suggesting that the product that is being used does not influence the outcome of the vaccination programme.

It would be necessary to analyze the reasons for not implementing a TM feeding period that covers the whole primary rota- and coronavirus susceptibility period for the calves in the future studies and to distinguish whether this is due to limited or confusing information in the vaccine instructions, arises from technical issues in TM collection and feeding or are there other aspects restricting the utilization of the maximal potential of a PVP.

### Association between pre-calving vaccination programme and calf mortality

The mortality rates in the V-period were higher among CEU farms than among CSU or ICU farms. Higher pre-vaccination mortality rates could derive from the relatively larger herd size in the CEU group, suggesting that the animals would receive less individual attention and would be prone to delayed detection of diseases, which can lead to higher mortality. On the other hand, larger herds might have somewhat higher threshold levels for making costly changes, such as implementation of a whole herd vaccination programme.

In three of the CEU group herds, a significant decrease in both the overall and diarrhea-induced mortality occurred during the V+ period when compared to the V- period. This implies that a large proportion of deaths were caused by diarrhea in these farms. While no significant change in calf age at death occurred in CEU farms during the V+ period, both the group-level and farm-level Kaplan-Meier survival graphs revealed that differences in mortality hazards between the two periods became evident after first week of life which is the highest risk period for calf scours due to rotavirus and coronavirus. This was different from the CSU farms, where the difference in calf mortality could be observed during the first week of calves’ life. These discrepancies indicate different disease patterns, risk factors and possibly different disease etiology in the herds of the two groups. It also suggests that the correct vaccination practices combined with prolonged TM feeding might help to delay the potentially lethal infection or alleviate the course of the disease for young calves.

In Farm 4 from the CEU group, no significant change in diarrhea-induced calf mortality was seen in the V+ period although the overall mortality decreased by almost 50%. Diarrhea was probably not the main cause of death for young calves in that farm and having the highest average age at death among all study farms also suggests that other problems in calf rearing might be more prevalent. According to registry data, “other reasons” was the second most common cause of calves exiting from this herd after “digestive disorders”. This can imply to the occurrence of multifactorial death cases that were difficult to categorize under only one disease or problem.

In Farm 7, a significant increase in diarrhea-induced calf mortality was detected during the V+ period, whereas the overall mortality increased slightly but nonsignificantly. In that farm, a known *Salmonella* Typhimurium outbreak occurred in the middle of the V+ period (data not shown) and was presumably the cause of such a severe deterioration in calf health and survival.

Calf mortality also increased in Farm 12 during the V+ period. Heifers were naturally mated in this farm and despite the continuous implementation of the PVP until this day, rotavirus was still diagnosed in some calves in summer 2020 (data not shown). Although the pregnancies were confirmed via rectal examination, some heifers might have been vaccinated too close to calving due to inaccurate determination of the insemination date, resulting in lower antibody titers in the colostrum and TM and subsequent weaker lactogenic protection of the calves. Another concern is that bovine rotavirus has many serogroups and serotypes based on its antigenic properties and group A rotaviruses with different G and P serotypes are the most common ones among calves [51]. The most prevalent group A rotavirus genotype reported in cattle worldwide is G6P[5], but the circulating genotypes might differ in herds that vaccinate against rotavirus and in herds that do not [51–53]. The genotypes used in BS and RC are G6P[1] and G6P[5], respectively [39, 40], and a difference in only the G or only the P gene could result in insufficient protection and clinical disease [54–57]. Virus genotyping was not performed in this study and further studies should analyze the epidemiology of rotavirus genotypes circulating in specific populations and take this into account when analyzing the efficacy of a PVP.

Among three CSU farms, the decrease in overall mortality was mostly derived from diarrhea-induced deaths although in one farm the decrease was not significant. In Farm 8 the changes in the mortality rates were not significant; however, this farm had an excellent situation regarding calf mortality even before the introduction of the PVP and the situation remained unchanged throughout the two study periods. The average age at death increased across the CSU farms which could suggest alleviation of the acute phase of the disease and imply that only diarrhea cases with longer disease course might have resulted with death.

In the ICU group, Farm 2 was very small and had very few death cases and in Farm 9 both the overall and diarrhea-induced mortality decreased, although nonsignificantly. However, in Farm 11, a significant and very drastic increase in calf mortality was seen in the V+ period and the majority of the increase could be attributed to diarrhea. Unfortunately, we do not have any additional information about this farm and can only speculate that there might have been an acute outbreak of a disease or a serious and sudden problem in calf rearing in general, but we are unable to draw any inferences explaining this increase. This extreme situation in Farm 11 is also accountable for the overall increase in CMH among the ICU farms.

### Farmers´ opinion about the efficacy of pre-calving vaccination programme

Most of the participating farmers had a subjective opinion that the incidence of diarrhea and calf mortality decreased during the first year of vaccination. Additionally, they generally thought that the implementation of a PVP is a cost-effective prophylactic measure. Interestingly, a decrease in morbidity and mortality was pointed out even in farms where either no significant change in mortality rates was seen or where the mortality rate even increased.

### Study limitations

Although only the herds that ear-tagged their calves within the first four days were included in the mortality analysis, the registry data might still miss some data regarding calves that died during those first four days. However, as the information was collected retrospectively, data from each farm was compared to earlier data from the same farm, and the practices regarding registration of births and deaths would most likely not change across the two-year study period, we consider the possible influence of this aspect to the study results negligible. Most likely, the analysis might have slightly underestimated the mortality rates in all study herds in both study periods.

The relatively small sample frame also set the limits to the study design. Despite a high number of calf records in the analysis, the number of study herds was small and we revealed a substantial herd effect. Although the herd effect was accounted for in the study design and analysis, more herds would be needed to extrapolate the results of this study.

The Estonian Livestock Performance Recording Ltd registry allows the farmer to state only one reason per animal upon its exit from the herd and we do not know if the stated reason was the only and most important reason of death. To our knowledge, the inaccuracy in producer diagnosis of mortality causes of calves has not been studied, and due to that we also assessed the change in overall mortality and assume that even in the case of misinterpretation of the reason of death or reporting bias by the farmer, a trend in overall mortality would still be visible. Also, the spectrum of diseases affecting neonatal dairy calves is rather small and with fairly understandable clinical manifestations.

One of the main limitations of this study is that the herds were not randomly allocated into study groups and that the farms used specific TM feeding practices due to reasons unknown to the researchers. Guided from the results of the study, we might assume that CSU farms experienced a positive effect of PVP during the first week of calves’ life whereas CEU herds had to extend the duration of TM feeding to the second week of calves´ life to achieve a noticeable on-farm effect. To bring more clarity into this, the effect of TM feeding practices on diarrhea-induced calf mortality should be studied using better controlled studies and also considering the disease-causing pathogens at the genotype level.

Another limitation is associated to the changes the farms might have made in addition to implementing the vaccination programme. In Farm 11 which was categorized as ICU farm, the feeding system for calves over nine days old was changed from hand-feeding to automatic feeder some time before initiating the PVP. No other major changes, e.g. building a new barn, pens or boxes for calves or implementing new keeping system or new feeding system occurred at the time of the initiation of the vaccination programme in any of the study herds that were included in the mortality analyses. However, as the data was collected retrospectively, we lack information about potential smaller changes or remedies that were implemented in the study farms along with the vaccination programme. Due to these reasons, we avoid making causal inferences between TM feeding practices and calf mortality. Nevertheless, as there were considerable differences in the association between calf mortality hazard in V- and V+ periods among correctly and incorrectly vaccinating herds, we suggest that correct implementation of a PVP can be considered as one possible tool in lowering diarrhea-induced calf mortality in large dairy farms.

## Conclusions

The present study revealed that pre-calving vaccination with a trivalent vaccine containing rotavirus, coronavirus and ETEC was mostly conducted in accordance with the vaccine manufacturers´ instructions; however, some farms neglected pregnant heifers from their pre-calving vaccination programme. According to the general recommendations and etiology of calf diarrhea, hyperimmune milk should be fed at least during the first two weeks of life, as calves are most susceptible to diarrheal pathogens at that time, and providing continuous lactogenic immunity is essential. In general, the veterinarians and farm managers that implemented vaccination programme in their herd had a positive experience regarding its impact on calf health and mortality. The latter was also confirmed by the data analysis identifying a significant drop in calf mortality rates in some of the farms, but herd effect was substantial. Further studies should aim to clarify the effect the duration of TM feeding has on calf mortality in better controlled conditions and formulate clearer instructions for the farmers regarding vaccinated cows’ milk feeding practices.

## Material and methods

### Study design and data collection

The present cohort study used retrospective information about vaccine usage and vaccinated cows´ milk feeding practices and compared calf-level mortality hazards in vaccination and pre-vaccination periods. A list of herds that implemented PVP against bovine rotavirus, bovine coronavirus and ETEC was obtained in August 2019 from the two largest veterinary pharmaceutical distributors known to be the main sellers of the vaccine in Estonia. Based on this information, all 15 dairy herds known to implement PVP were enrolled in the study. For all recruited herds, animal-level registry data was obtained from the Estonian Agricultural Registers and Information Board containing information about the date of birth, selling, slaughter, death or euthanasia of all ear-tagged animals in the herds within the pre-determined period. Registry data regarding the farmers’ stated reasons of leaving the herd was acquired from the Estonian Livestock Performance Recording Ltd. In order to ascertain the reason for each death, data from the two registries were merged.

A questionnaire was composed that included questions about the incentive for vaccination, diarrhea pathogen diagnostics before the start of the PVP, vaccination procedures (which animals received the vaccine, who performed the vaccination, at which time during the lactation cycle was the vaccination performed, which vaccine was used and into which body region was the vaccine administered), colostrum and pooled transition milk (cow´s milk in the first four post-partum days, TM) feeding management, the respondent´s subjective opinion about the effect of the PVP on calf diarrhea incidence and mortality and the possible cost-effectiveness of the PVP. The exact starting date of the vaccination programme, calves’ age at ear-tagging and facility numbers in which the calves were housed were also specified in the questionnaire along with information whether any major changes, e.g. building a new barn, pens or boxes for calves or implementing new keeping system or new feeding system occurred at the time of the initiation of the vaccination programme. The majority of the questions were open-ended or multiple-choice questions with the latter always including an answer category “Other” for unique answers or specifications. The questionnaire was pretested in two non-vaccinating farms to understand whether the questions were understandable and answer categories exhaustive. In 12 farms, the questionnaire was filled during a farm visit and in three farms, a phone interview was carried out. The interviewee was the farm veterinarian or the farm manager in all cases. All interviews were carried out by one of the two pretrained persons and were conducted between September 2019 and March 2020.

### Assignment of herds to study groups

During the last five years, two oil adjuvanted products containing bovine rotavirus, bovine coronavirus and enterotoxigenic *E. coli* (F5 antigen) have been available in Estonia for PV: Bovigen Scour (Virbac, France) and Rotavec Corona (Manufacturer: MSD Animal Health, Republic of Ireland; marketing authorization holder: Intervet International BV, Netherlands). The summary of product characteristics (SPC) of both of these vaccines states that a single dose of the vaccine should be administered during each pregnancy, 3-12 weeks before expected calving [39, 40] and farms were categorized based on compliance with vaccination instructions. As the susceptibility of calves to rotavirus, coronavirus and ETEC infections is highest during the first two weeks of life (Cho and Yoon, 2014), farms were further categorized based on the length of TM feeding. To analyze the association between implementing the PVP and dairy calf mortality, the farms were allocated into three groups based on the two criteria and the information provided via the questionnaire.

To qualify as a complete extended PVP user (CEU), the farm had to vaccinate all cows and pregnant heifers 3-12 weeks before the expected calving and feed the calves TM from vaccinated cows at least during the first two weeks of calves´ life. Farms that vaccinated all cows and pregnant heifers 3-12 weeks before the expected calving and fed the calves TM for less than 14 days were categorized as complete standard PVP users (CSU). Farms that did not follow the vaccination instructions and fed the calves TM for less than 14 days were grouped as incomplete PVP users (ICU) in this study.

### Statistical analysis

**Fig. 3:**
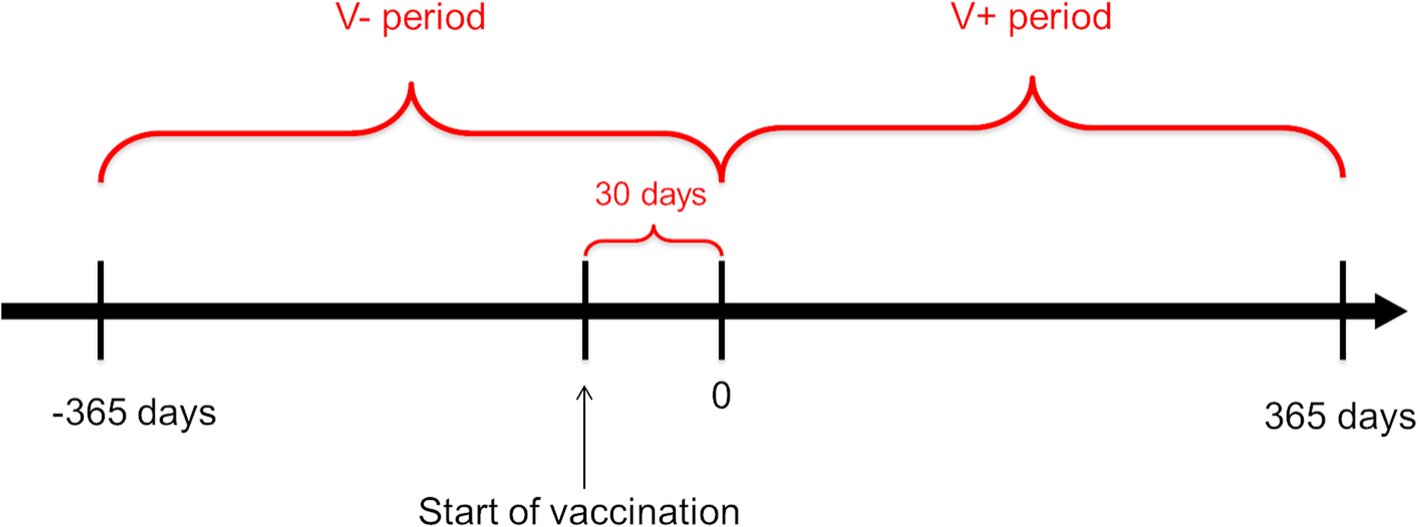
Determination of the herd-level study periods discriminating pre-vaccination (V-) period and first year of vaccination (V+) period

To analyze the association between PVP and calf mortality hazard, survival analysis was performed in which the herd-level calf mortality rates were compared in the pre-vaccination period (V- period) and during the first year of vaccination (V+ period) in each herd. As it is suggested that vaccination should be performed not later than three weeks before the expected calving and considering the fact that not all calvings happen at the predicted time, a waiting period of 30 days after the start of the vaccination was applied, meaning that the V+ period started one month after the farm started vaccinating their animals and lasted for 365 days. The V- period was determined as exactly one year prior to the V+ period (Fig. 3).

Data was used and handled as time-to-event data (´*stset*´ command) in Stata MP14 (College Station, TX: StataCorp LP). The calf-level observation period started with the birth of the calf or at the beginning of the observation period for calves that were born before the start of the study period to account for left truncation [58]. As the incidence of rota- and coronavirus infections peaks at two weeks of age [7, 9], but possible death might not occur immediately after becoming infected, the calf-level observation period lasted for 21 days. Two models were fitted for each study farm with the first analyzing the changes in overall calf-level mortality where the observation period ended with either on-farm mortality (unassisted death or euthanasia being the event of interest) or right censoring due to selling, slaughter, reaching 21 days of age or at the end of the study period. A second model was made to exclusively examine the diarrhea-induced calf mortality in the two periods. For the second model the observation period ended with either diarrhea-induced on-farm mortality (unassisted death or euthanasia due to diarrhea being the event of interest) or right censoring due to on-farm mortality caused by other reasons than diarrhea or selling, slaughter, reaching 21 days of age or at the end of the study period. As a background information, herd-level calf mortality rates were calculated for each farm as the number of deaths per 100 calf-months in the V- and V+ periods in total (including all mortality cases unrelated to farmers´ stated reason) and due to diarrhea (included farmers´ stated reasons “digestive disorders” and “metabolic disorders”).

Estonian farmers are obliged to ear-tag calves during the first 20 days of age [59]; therefore, the registry data might miss information about deaths that occurred during the first weeks of calves’ life. To overcome this problem in the analysis in which the association between implementing PVP and calf on-farm mortality was analyzed, only farms that stated they ear-tag their calves within the first four days after birth were included. Although the use of PV and calf feeding practices were analyzed using data from all 15 recruited farms, two CSU herds were excluded from the mortality analyses due to this restriction. Kaplan-Meier survival graphs (´*sts graph*´ command) discriminating survival curves for V- and V+ periods were composed across all CEU (*n* = 6), CSU (*n* = 4) and ICU (*n* = 3) farms. Mixed-effect Cox regression analysis (´*stcox*´ command) with herd included as random effect (specified as option ´*shared*´) was used to analyze the statistical difference between calf mortality hazards in V- and V+ periods (specified with dichotomous fixed-effect variable ´*period*´) in all CEU, CSU and ICU farms. The association between the PVP and the age at death was analyzed using a random effect linear regression model specifying age at death as the outcome variable, study period as a fixed effect and herd as a random effect, calculated separately for CEU, CSU and ICU herds.

## Supplementary Information

**Additional file 1: Supplementary Figure 1.** Kaplan-Meier survival graphs presenting the diarrhea-induced mortality probabilities of calves up to 21 days of age in six Estonian dairy herds using a pre-calving vaccination programme. Farms vaccinated all cattle 3-12 weeks before the expected calving and fed the calves with vaccinated cows´ milk from the first four post-partum days at least during the first two weeks of calves´ life. The red and blue lines represent diarrhea-induced calf mortality during the first year of vaccination and the year before implementation of the vaccination programme, respectively, together with 95% confidence intervals as the shaded areas.

**Additional file 2: Supplementary Figure 2.** Kaplan-Meier survival graphs presenting the diarrhea-induced mortality probabilities of calves up to 21 days of age in four Estonian dairy herds using a pre-calving vaccination programme. Farms vaccinated all cattle 3-12 weeks before the expected calving but fed the calves with vaccinated cows´ milk from the first four post-partum days for less than 14 days. The red and blue lines represent diarrhea-induced calf mortality during the first year of vaccination and the year before implementation of the vaccination programme, respectively, together with 95% confidence intervals as the shaded areas.

**Additional file 3: Supplementary Figure 3.** Kaplan-Meier survival graphs presenting the diarrhea-induced mortality probabilities of calves up to 21 days of age in three Estonian dairy herds using a pre-calving vaccination programme that do not vaccinate all of their cattle 3-12 weeks before the expected calving. The red and blue lines represent diarrhea-induced calf mortality during the first year of vaccination and the year before implementation of the vaccination programme, respectively, together with 95% confidence intervals as the shaded areas.

## Data Availability

Data used in the present study was obtained from Estonian national registries Estonian Agricultural Registers and Information Board and Estonian Livestock Performance Recording Ltd under a confidentiality agreement and is not allowed to be made publicly accessible.

## Abbreviations

CEU: Complete extended pre-calving vaccination programme user
CMH: Calf mortality hazard
CSU: Complete standard pre-calving vaccination programme user
ETEC: Enterotoxigenic *Escherichia coli*
HR: Hazard rate ratio
ICU: Incomplete pre-calving vaccination programme user
PV: Pre-calving vaccination
PVP: Pre-calving vaccination programme
SPC: Summary of product characteristics
TM: Transition milk (cow´s milk in the first four post-partum days)
V- period: Pre-vaccination period
V+ period: First year of vaccination
