# Supplementary material for: Implementation of a pre-calving vaccination programme against rotavirus, coronavirus and enterotoxigenic Escherichia coli (F5) and association with dairy calf survival
Source: BMC Vet Res. 2022 Jan 28;18:59. doi: 10.1186/s12917-022-03154-2 (PMC8935617; doi:10.1186/s12917-022-03154-2)

Supplementary Figure 1. Kaplan-Meier survival graphs presenting the diarrhea-induced mortality probabilities of calves up to 21 days of age in six Estonian dairy herds using a pre-calving vaccination programme. Farms vaccinated all cattle 3-12 weeks before the expected calving and fed the calves with vaccinated cow´s milk from the first four post-partum days at least during the first two weeks of calves´ life. The red and blue lines represent diarrhea-induced calf mortality during the first year of vaccination and the year before implementation of the vaccination programme, respectively, together with 95% confidence intervals as the shaded areas.


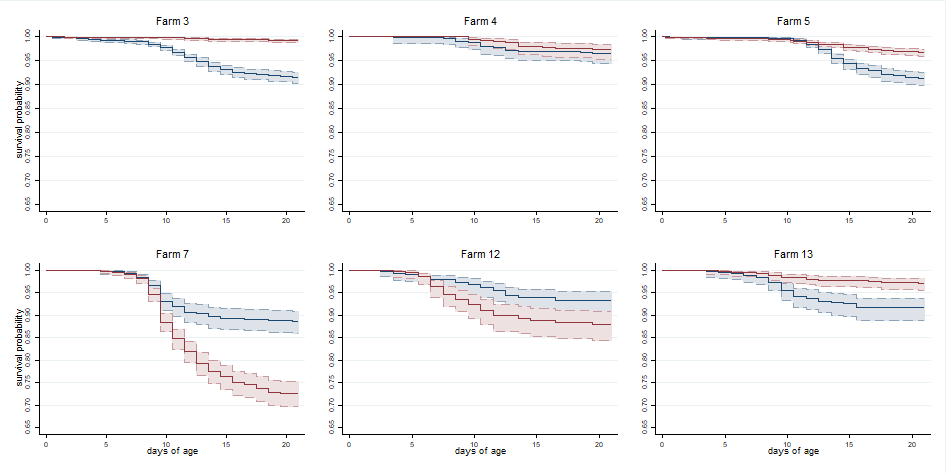

Supplement: Supplementary file 1 — Additional file 1: Supplementary Figure 1. Kaplan-Meier survival graphs presenting the diarrhea-induced mortality probabilities of calves up to 21 days of age in six Estonian dairy herds using a pre-calving vaccination programme. Farms vaccinated all cattle 3-12 weeks before the expected calving and fed the calves with vaccinated cows´ milk from the first four post-partum days at least during the first two weeks of calves´ life. The red and blue lines represent diarrhea-induced calf mortality during the first year of vaccination and the year before implementation of the vaccination programme, respectively, together with 95% confidence intervals as the shaded areas. [file 12917_2022_3154_MOESM1_ESM.docx]
